# Splenectomy Correlates With Increased Risk of Pyogenic Liver Abscess: A Nationwide Cohort Study in Taiwan

**DOI:** 10.2188/jea.JE20140267

**Published:** 2015-09-05

**Authors:** Shih-Wei Lai, Hsueh-Chou Lai, Cheng-Li Lin, Kuan-Fu Liao

**Affiliations:** 1 College of Medicine, China Medical University, Taichung, Taiwan; 2 Department of Family Medicine, China Medical University Hospital, Taichung, Taiwan; 3 College of Chinese Medicine, China Medical University, Taichung, Taiwan; 4 Division of Hepato-gastroenterology, Department of Internal Medicine, China Medical University Hospital, Taichung, Taiwan; 5 Management Office for Health Data, China Medical University Hospital, Taichung, Taiwan; 6 Graduate Institute of Integrated Medicine, China Medical University, Taichung, Taiwan; 7 Department of Internal Medicine, Taichung Tzu Chi General Hospital, Taichung, Taiwan

**Keywords:** alcoholism, diabetes mellitus, pyogenic liver abscess, splenectomy

## Abstract

**Objectives:**

Little is known about the risk of pyogenic liver abscess in patients with splenectomy. We explored the relationship between splenectomy and pyogenic liver abscess in Taiwan.

**Methods:**

We conducted a nationwide cohort analysis using the hospitalization dataset of the Taiwan National Health Insurance Program. We included 17 779 subjects aged 20–84 years who underwent splenectomy in 1998 to 2010 (splenectomy group) and 70 855 randomly selected subjects without splenectomy (non-splenectomy group). Both groups were matched by sex, age, other comorbidities, and hospitalization year of receiving splenectomy. The incidence of pyogenic liver abscess at the end of 2011 was measured. The multivariable Cox proportional hazard regression model was used to estimate the hazard ratios and 95% confidence intervals for pyogenic liver abscess associated with splenectomy and other comorbidities.

**Results:**

The overall incidence rate was 3.75-fold higher in the splenectomy group than that in the non-splenectomy group (2.15 vs 0.57 per 1000 person-years; 95% confidence interval, 3.57–3.94). After controlling for potential confounding factors, the adjusted hazard ratio of pyogenic liver abscess was 3.89 in subjects with splenectomy (95% confidence interval, 3.20–4.72) when compared with subjects without splenectomy. In further analysis, the hazard ratio markedly increased to 14.34 for those with splenectomy and having any of the assessed comorbidities, including alcoholism, biliary stone, chronic kidney disease, chronic liver diseases, and diabetes mellitus (95% confidence interval, 10.61–19.39).

**Conclusions:**

Patients with splenectomy are at an increased risk of developing pyogenic liver abscess, particularly when they have comorbid conditions.

## INTRODUCTION

The human spleen mainly serves an immune function against invading microorganisms.^1^^,^^2^ Patients with splenectomy are more likely than those without splenectomy to suffer severe life-threatening infections, which are recognized as overwhelming postsplenectomy infections with substantial morbidity and mortality.^3^^,^^4^ Recently, there is growing evidence that patients with splenectomy are at increased risk of pulmonary tuberculosis and type II diabetes mellitus,^5^^,^^6^ but risk of pyogenic liver abscess has not yet been examined.

Pyogenic liver abscess is an infective disease of the liver, which can be caused by numerous microorganisms. The overall mortality rate of pyogenic liver abscess ranges from 10% to 25%, depending on the population studied.^7^^,^^8^ An increasing amount of literature reveals that numerous risk factors, such as biliary tract disease, cirrhosis, diabetes mellitus, male sex, cancer, and renal disease, are associated with pyogenic liver abscess,^8^^–^^12^ but splenectomy has not yet been evaluated.

We rationally hypothesize an association between splenectomy and pyogenic liver abscess due to the immunocompromised condition caused by splenectomy, which can further increase the risk of microorganism invasion of the liver. However, published literature from epidemiological studies on this issue is scarce. Given that splenectomy is associated with overwhelming postsplenectomy infections and pyogenic liver abscess carries a potential fatality, exploring the risk of pyogenic liver abscess in patients with splenectomy may have significant clinical and public health implications. Therefore, to explore whether there is an association between splenectomy and pyogenic liver abscess, we conducted a nationwide cohort study using the hospitalization dataset of the Taiwan National Health Insurance Program.

## METHODS

### Data source

We conducted a nationwide cohort study using the National Health Insurance Research Database (NHIRD) released by the Taiwan National Health Research Institutes. In March 1995, the National Health Insurance Program was initiated in Taiwan to provide comprehensive medical care to the public. According to the annual statistic report of the National Health Insurance Program, the coverage rate was nearly 99% of the entire 23 million people living in Taiwan.^13^ A committee at the Bureau of National Health Insurance was responsible for randomly selecting claims and checking the accuracy of claims. The NHIRD encrypts patients’ personal information to protect privacy and provides researchers with anonymous identification numbers associated with relevant claim information, including patient’s sex, date of birth, medical services utilized, and prescriptions. We identified the investigated diseases based on the International Classification of Diseases, Ninth Revision, Clinical Modification (ICD-9-CM) codes. This study was approved by the Institutional Review Board at China Medical University and Hospital in Taiwan (CMU-REC-101-012).

### Sampled participants

Using the inpatient claim dataset of the NHIRD, all hospitalized subjects aged 20–84 who received splenectomy (ICD-9-CM procedure code 41.5) between January 1, 1998, and December 31, 2010, were identified as the splenectomy group. The date for receiving splenectomy was defined as the index date. For each subject with splenectomy, four subjects without splenectomy were randomly selected from the NHIRD as the non-splenectomy group. Both groups were matched by sex, age (every 5-year span), other comorbidities, and the hospitalization year of receiving splenectomy. Subjects with history of pyogenic liver abscess, amebic liver abscess, or liver transplantation before the index date were excluded from this study (Figure).

**Figure.**
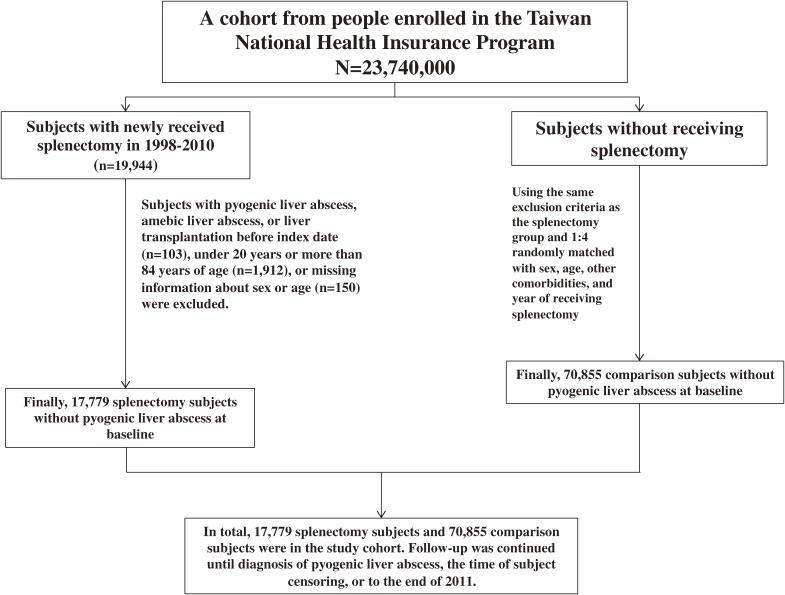
Flow chart depicting the selection process for study participants.

### Outcome and comorbidities

The main outcome was a new diagnosis of pyogenic liver abscess based on hospital discharge registries during the follow-up period. Each subject was monitored from the index date until being diagnosed with pyogenic liver abscess; being censored because of loss to follow-up, death, or withdrawal from insurance; or the end of December 31, 2011.

We selected the following comorbidities potentially associated with pyogenic liver abscess: alcoholism, biliary stone, chronic kidney disease, diabetes mellitus, and chronic liver diseases, which included cirrhosis, alcoholic liver damage, hepatitis B, hepatitis C, and other chronic hepatitis. All comorbidities were assessed using ICD-9-CM codes (eTable 1).

### Statistical analysis

Differences in sex, age, and baseline comorbidities between the splenectomy and the non-splenectomy group were compared using the Chi-square test for categorical variables and the *t* test for continuous variables. Follow-up time (in person-years) was used to estimate the incidence rate and incidence rate ratio (IRR) with 95% confidence interval (CI) of splenectomy group to non-splenectomy group using Poisson regression, by sex, age, and follow-up period. The multivariable Cox proportional hazards regression model was used to estimate the hazard ratio (HR) with 95% CI of pyogenic liver abscess associated with splenectomy and other comorbidities after simultaneously controlling for variables found to be significant in the univariable Cox proportional hazard regression model. To address the concern of constant proportionality, we examined the proportional hazard model assumption using a test of scaled Schoenfeld residuals. In the model evaluating pyogenic liver abscess risk throughout the overall follow-up period, results of the test revealed a significant relationship between Schoenfeld residuals for splenectomy and follow-up time, suggesting the proportionality assumption was violated (*P* < 0.001). In the subsequent analyses, we stratified the follow-up duration to deal with the violation of proportional hazard assumption. All statistical analyses were performed using SAS 9.3 (SAS Institute, Cary, NC, USA). Two-tailed *P* < 0.05 was considered statistically significant.

## RESULTS

### Baseline information of the study population

Table 1 reveals the baseline information of the study population. There were 17 779 subjects with splenectomy and 70 855 subjects without splenectomy during the study period, with similar distribution in sex and age. Male subjects accounted for 62.4% of the study population. The mean (standard deviation [SD]) age of the splenectomy group was 53.6 (17.7) years, and that of the non-splenectomy group was 53.3 (17.8) years, with 42.3% of all subjects aged <40 years. The mean (SD) follow-up period was 4.48 (4.05) years in the splenectomy group and 6.46 (3.87) years in the non-splenectomy group. There was no significant difference in the prevalence of comorbidities studied between the splenectomy group and the non-splenectomy group (Chi-square test, *P* > 0.05 for all).

**Table 1.** Baseline information between splenectomy group and non-splenectomy group

Variable	Splenectomy *n* = 17 779		Non-splenectomy *n* = 70 855		*P* value^a^
	
	*n*	(%)	*n*	(%)	
Sex					0.99
Male	11 097	(62.4)	44 219	(62.4)	
Female	6682	(37.6)	26 636	(37.6)	
Age group, years					0.99
20–39	7512	(42.3)	29 884	(42.2)	
40–64	4813	(27.1)	19 180	(27.1)	
65–84	5454	(30.7)	21 791	(30.8)	
Baseline comorbidities before index date					
Alcoholism	541	(3.04)	2058	(2.90)	0.33
Biliary stone	1120	(6.30)	4347	(6.14)	0.42
Chronic kidney disease	351	(1.97)	1310	(1.85)	0.27
Chronic liver diseases	3732	(21.0)	14 672	(20.7)	0.40
Diabetes mellitus	1967	(11.1)	7741	(10.9)	0.60

Data are presented as the number of subjects in each group, with percentages given in parentheses.

^a^Chi-square test comparing subjects with and without splenectomy.

### Incidence of pyogenic liver abscess stratified by sex, age, and follow-up period

Table 2 reveals the incidence rates of pyogenic liver abscess. At the end of the cohort study, the overall incidence rate was 3.75-fold higher in the splenectomy group than in the non-splenectomy group (2.15 vs 0.57 per 1000 person-years; 95% CI, 3.57–3.94). The incidence rates of pyogenic liver abscess, as stratified by sex, age, and follow-up period, were all higher in the splenectomy group than in the non-splenectomy group. The incidence rates of pyogenic liver abscess increased with age in both groups, with the highest incidence rates in the splenectomy group aged 65–84 years. Stratified analysis by follow-up period revealed that the incidence rate of pyogenic liver abscess decreased with follow-up time in the splenectomy group. The IRR of pyogenic liver abscess was significantly increased in the first year of follow-up (IRR 8.40; 95% CI, 7.97–8.85) and subsequently decreased with increasingly longer follow-up, to 2.40 for 5 years of follow-up. The risk remained for more than 5 years of follow-up. In the analysis on the entire follow-up period, the proportional hazard assumption was not met. These observations consistently suggested a time-varying association between splenectomy and pyogenic liver abscess, particularly within the first year after splenectomy.

**Table 2.** Incidence density of pyogenic liver abscess stratified by sex, age and follow-up period between splenectomy group and non-splenectomy group

Variable	Splenectomy				Non-splenectomy				IRR (95% CI)
	
	*n*	Event	Person-years	Incidence^a^	*n*	Event	Person-years	Incidence^a^	
All	17 779	171	79 669	2.15	70 855	262	457 798	0.57	3.75 (3.57–3.94)
Sex									
Male	11 097	106	48 677	2.18	44 219	164	284 391	0.58	3.78 (3.55–4.01)
Female	6682	65	30 993	2.10	26 636	98	173 407	0.57	3.71 (3.43–4.01)
Age group, years									
20–39	7512	59	43 809	1.35	29 884	69	211 477	0.33	4.13 (3.83–4.45)
40–64	4813	59	19 769	2.98	19 180	91	121 908	0.75	4.00 (3.65–4.38)
65–84	5454	53	16 092	3.29	21 791	102	124 414	0.82	4.02 (3.68–4.38)
Follow-up period									
≤1 year	17 779	82	14 697	5.58	70 855	46	69 252	0.66	8.40 (7.97–8.85)
2–3 years	13 254	35	22 004	1.59	67 766	70	120 702	0.58	2.74 (2.59–2.90)
4–5 years	9217	34	27 430	1.24	53 353	86	166 703	0.52	2.40 (2.25–2.57)
>5 years	4864	20	15 538	1.29	30 696	60	101 141	0.59	2.17 (1.99–2.37)

CI, confidence interval; IRR, incidence rate ratio (splenectomy vs non-splenectomy).

^a^Incidence: per 1000 person-years.

### Hazard ratio of pyogenic liver abscess associated with splenectomy

Table 3 reveals the HR of pyogenic liver abscess associated with splenectomy and other comorbidities. Only those found to be significant in the univariable analysis were further examined in the multivariable analysis. After controlling for age, alcoholism, biliary stone, chronic liver diseases, chronic kidney disease, and diabetes mellitus, the multivariable Cox proportional hazards regression model revealed that the adjusted HR of pyogenic liver abscess was 3.89 (95% CI, 3.20–4.72) for the splenectomy group when compared with the non-splenectomy group. Alcoholism (adjusted HR 1.77; 95% CI, 1.17–2.67), biliary stone (adjusted HR 2.19; 95% CI, 1.69–2.84), chronic kidney disease (adjusted HR 1.93; 95% CI, 1.18–3.15), chronic liver diseases (adjusted HR 2.50; 95% CI, 2.02–3.09) and diabetes mellitus (adjusted HR 1.74; 95% CI, 1.37–2.22) were also associated with pyogenic liver abscess. Every 1-year increase in age was associated with a 1.03-fold increased risk of developing pyogenic liver abscess (95% CI, 1.02–1.03).

**Table 3.** Adjusted hazard ratio and 95% confidence interval of pyogenic liver abscess associated with splenectomy and other comorbidities

Variable	Crude		Adjusted^a^	
	
	HR	(95% CI)	HR	(95% CI)
Age, per year	1.02	(1.02–1.03)	1.03	(1.02–1.03)
Baseline comorbidities before index date, yes vs no				
Splenectomy	3.60	(2.97–4.37)	3.89	(3.20–4.72)
Alcoholism	2.68	(1.82–3.96)	1.77	(1.17–2.67)
Biliary stone	3.80	(2.96–4.87)	2.19	(1.69–2.84)
Chronic kidney disease	3.14	(1.93–5.10)	1.93	(1.18–3.15)
Chronic liver diseases	3.35	(2.86–4.06)	2.50	(2.02–3.09)
Diabetes mellitus	3.00	(2.38–3.77)	1.74	(1.37–2.22)

^a^Adjusted for age, alcoholism, biliary stone, chronic kidney disease, chronic liver diseases and diabetes mellitus.

### Interaction effect on risk of pyogenic liver abscess between splenectomy and comorbidities

Table 4 reveals the interaction effect on risk of pyogenic liver abscess between splenectomy and other comorbidities, including alcoholism, biliary stone, chronic kidney disease, chronic liver diseases, and diabetes mellitus. Compared to subjects without splenectomy or comorbidity, the adjusted HR of pyogenic liver abscess was 5.84 (95% CI, 4.37–7.81) for subjects with splenectomy alone but without comorbidity. The HR was 5.01 (95% CI, 3.89–6.47) for subjects with any comorbidity but without splenectomy. The HR markedly increased to 14.34 (95% CI, 10.61–19.39) for those with splenectomy and any comorbidity (*P* value for interaction <0.001).

**Table 4.** Interaction effect on pyogenic liver abscess between splenectomy and other comorbidities

Variable		*n*	Event	Person-years	Incidence^a^	Adjusted HR^b^ (95% CI)
Splenectomy	Comorbidity^c^					
No	No	49 400	93	345 670	0.27	1 (Reference)
No	Yes	21 455	169	112 128	1.51	5.01 (3.89–6.47)
Yes	No	12 350	91	61 082	1.49	5.84 (4.37–7.81)
Yes	Yes	5429	80	18 587	4.30	14.34 (10.61–19.39)

CI, confidence interval; HR, hazard ratio.

^a^Incidence: per 1000 person-years.

^b^Adjusted for age.

^c^Patients with any examined comorbidities, including alcoholism, biliary stone, chronic kidney disease, chronic liver diseases and diabetes mellitus, were classified as the comorbidity group.

The interaction between splenectomy and comorbidities was significant (*P* value for interaction <0.001).

## DISCUSSION

In this nationwide cohort study, we found that the incidence rate of pyogenic liver abscess among patients with splenectomy was slightly higher than that reported in Tsai et al’s study among patients in Taiwan with end-stage renal disease (21.5 vs 18.2 per 10 000 person-years)^11^ but was significantly higher than that reported in Molle et al’s study among patients in Denmark with cirrhosis (215 vs 23.3 per 100 000 person-years).^9^ Whether this is due to differences in patient-finding strategy or because patients with splenectomy have a substantially higher risk of developing pyogenic liver abscess needs further research to clarify.

In our study, after controlling for potential confounding factors, we also observed that patients with splenectomy were at increased risk of pyogenic liver abscess (adjusted HR 3.89). The HR seems to be higher than that observed for other comorbidities. The HR was not confounded by other studied comorbidities because there was no significant difference in the prevalence of comorbidities between the splenectomy group and the non-splenectomy group, so the increased risk of pyogenic liver abscess in patients with splenectomy cannot be totally attributable to the effect of comorbidities. In further analysis, even in the absence of any comorbidity, patients with splenectomy still had a higher risk of pyogenic liver abscess than those without splenectomy (adjusted HR 5.84). This indicates splenectomy may have a unique role on risk of pyogenic liver abscess independent of other comorbidities. These findings are compatible with the literature showing that patients with splenectomy are not only more prone than those without splenectomy to suffer severe life-threatening infection due to the immunocompromised condition caused by splenectomy^3^^,^^4^ but are also at an increased risk of developing pyogenic liver abscess.

Extensive evidence has revealed that the human spleen mainly serves a protective role against invading microorganisms on the basis of bactericidal capacity of lymphoid cells and macrophages and the humoral immune response.^14^^–^^17^ Upon removal of the spleen, normal immune functions, such as phagocytic activities and the humoral immune response, may be significantly changed. Therefore, impaired immune functions after splenectomy may increase the risk of life-threatening infection and pyogenic liver abscess.

Some important caveats in this present study deserve discussion. First, the underlying causes for splenectomy in this present study were not recorded due to the inherent limitation of this insurance database. For example, hematological malignant diseases may require splenectomy in some patients. Cancer is also associated with pyogenic liver abscess.^8^^,^^10^ In fact, we observed that cancer was the most common diagnosis accompanying splenectomy at the time of discharge in the present study. In further analysis, compared to patients without splenectomy, the adjusted HR of pyogenic liver abscess was 5.42 (95% CI, 4.14–7.10) for patients with splenectomy and with cancer (data not shown in table). Therefore, whether the risk of pyogenic liver abscess can be attributable to splenectomy alone or is confounded by the underlying causes for splenectomy is a critical point meriting further clarification. Second, due to the same limitation, the exact causes of pyogenic liver abscess in the present study were not recorded. According to our data, alcoholism, biliary stone, chronic kidney disease, chronic liver diseases, and diabetes mellitus may be other factors related to pyogenic liver abscess. Third, the adjusted HR of pyogenic liver abscess was 5.84 for patients with splenectomy alone, and the adjusted HR markedly increased to 14.34 for patients with splenectomy and any of the comorbidities, including alcoholism, biliary stone, chronic kidney disease, chronic liver diseases, and diabetes mellitus. Fourth, the highest risk of pyogenic liver abscess was noted within the first year after splenectomy. Although the risk gradually decreased over time, the risk remained for more than 5 years after splenectomy. These findings are compatible with previous studies showing that the majority of severe infections occur within the first 3 years after splenectomy, and, although the risk declines over time, the risk might last for more than 5 years after splenectomy.^18^^–^^20^ We think that the impaired immune functions might be more significant within the first year after splenectomy, which more likely precipitates development of pyogenic liver abscess and other severe infections. Therefore, when patients present with typical features of pyogenic liver abscess, including fever, right upper abdominal pain, and body weight loss,^12^ clinicians should strongly suspect the probability of pyogenic liver abscess among patients within the first year after splenectomy, particularly when they have other comorbidities.

The strength of this study is that we used a hospitalization dataset with a large sample size and great statistical power. The diagnosis codes of included comorbidities have been documented in previous studies.^21^^,^^22^ In addition, the topic is novel and the results are fascinating, and the study design, statistical methods, and limitations are described in detail. The results suggest some clinical importance and contribute to understanding of pyogenic liver abscess. Our results were relatively convincing because the splenectomy group and the non-splenectomy group had similar distributions of studied comorbidities. Therefore, the confounding effects of these comorbidities on risk of pyogenic liver abscess should be minimal.

In conclusion, patients with splenectomy are at an increased risk of developing pyogenic liver abscess compared to those without splenectomy, particularly when splenectomy patients also have other comorbid conditions, including alcoholism, biliary stone, chronic kidney disease, chronic liver diseases, and diabetes mellitus.

## ONLINE ONLY MATERIAL

eTable 1. Comorbidities examined in the study.
