# Supplementary material for: Splenectomy Correlates With Increased Risk of Pyogenic Liver Abscess: A Nationwide Cohort Study in Taiwan
Source: J Epidemiol. 2015 Sep 5;25(9):561–6. doi: 10.2188/jea.JE20140267 (PMC4549607; doi:10.2188/jea.JE20140267)
Supplement: eTable 1. [file je-25-561-s001.pdf]

eTable 1. Comorbidities examined in the study

Comorbidities	ICD-9-CM code
Alcoholism	291, 303, 305.00, 305.01, 305.02, 305.03, 790.3 and V11.3
Amebic liver abscess	006.3
Biliary stone	574
Chronic kidney diseases	585–586 and 588.8–588.9
Chronic liver diseases, including cirrhosis, alcoholic liver damage, hepatitis B, hepatitis C, and other chronic hepatitis	571.2, 571.5, 571.6, 571.0, 571.1, 571.3, V02.61, 070.20, 070.22, 070.30, 070.32, V02.62, 070.41, 070.44, 070.51, 070.54, 571.40, 571.41, 571.49, 571.8 and 571.9
Diabetes mellitus	250
Liver transplantation	996.82 and V42.7
Pyogenic liver abscess	572.0
Splenectomy	procedure code 41.5

ICD-9-CM, International Classification of Diseases, Ninth Revision, Clinical Modification.
